# Choosing a laser for laser speckle contrast imaging

**DOI:** 10.1038/s41598-019-39137-x

**Published:** 2019-02-22

**Authors:** Dmitry D. Postnov, Xiaojun Cheng, Sefik Evren Erdener, David A. Boas

**Affiliations:** 1 Boston University, Department of Biomedical Engineering, Boston, MA 02215 USA; 2 Copenhagen University, Department of Biomedical Sciences, Copenhagen, 2200 Denmark

## Abstract

The use of laser speckle contrast imaging (LSCI) has expanded rapidly for characterizing the motion of scattering particles. Speckle contrast is related to the dynamics of the scattering particles via a temporal autocorrelation function, but the quality of various elements of the imaging system can adversely affect the quality of the signal recorded by LSCI. While it is known that the laser coherence affects the speckle contrast, it is generally neglected in *in vivo* LSCI studies and was not thoroughly addressed in a practical matter. In this work, we address the question of how the spectral width of the light source affects the speckle contrast both experimentally and through numerical simulations. We show that commonly used semiconductor laser diodes have a larger than desired spectral width that results in a significantly reduced speckle contrast compared with ideal narrow band lasers. This results in a reduced signal-to-noise ratio for estimating changes in the motion of scattering particles. We suggest using a volume holographic grating stabilized laser diode or other diodes that have a spectrum of emitted light narrower than ≈1 nm to improve the speckle contrast.

## Introduction

Laser speckle contrast imaging (LSCI), through a simple camera exposure, provides a rapid wide field characterization of the motion of light scattering particles^1^. Over the past 15 years, it has become  widely used as a blood flow imaging tool, where it has been applied for measuring blood flow in the brain^2^, skin^1^, retina^3^, mesenterium^4^ and kidney^5^.

The technique is based on the analysis of the imaged speckle pattern that arises from random interference of coherent light scattered by a media comprised of light scattering particles. Motion of the scattering particles results in fluctuations of the interference pattern, which can be recorded as intensity variations by a camera. The temporal and spatial statistics of the recorded speckle pattern contains information about the motion. In particular, due to the finite integration time of the camera, motion will cause blurring of the speckle pattern, reducing the speckle contrast.

The most common way to quantify the blurring is to calculate local speckle contrast^1,2^, which can be related to speed via field and intensity autocorrelation functions^2^. Theoretically, the contrast of the polarized speckle pattern takes values between 0 and 1^6^, where 0 means that scatterers are fast enough to completely blur the speckle pattern, while 1 corresponds to a static, fully developed speckle pattern. In practice, however, the maximum speckle contrast depends on the parameters of the imaging system that generally reduce the range of achievable contrast values, affecting the signal-to-noise ratio and sensitivity. The optimal exposure time^7^, neighborhood and speckle size^6^ have been determined. Kirkpatrick *et al*.^8^ have demonstrated that insufficient spatial sampling of the speckle leads to a decreased contrast value. Speckle studies from almost 50 years ago have shown that source with partial coherence will produce speckle pattern with decreased contrast^9,10^. The effect, however, did not seem to be significant for laser diodes which have relatively long (>100 *μ*m) coherence length, thus was generally neglected in LSCI studies. Although previously we have demonstrated that the optical power density plays a significant role in the signal-to-noise ratio^11^, the impact of the coherence properties of semiconductor laser diodes traditionally utilized for LSCI has not been explored.

Here, we approach the question of how the light source spectral width affects the speckle pattern and what are the consequences for *in vivo* laser speckle imaging.

## Results

### Static speckle

**Figure 1:**
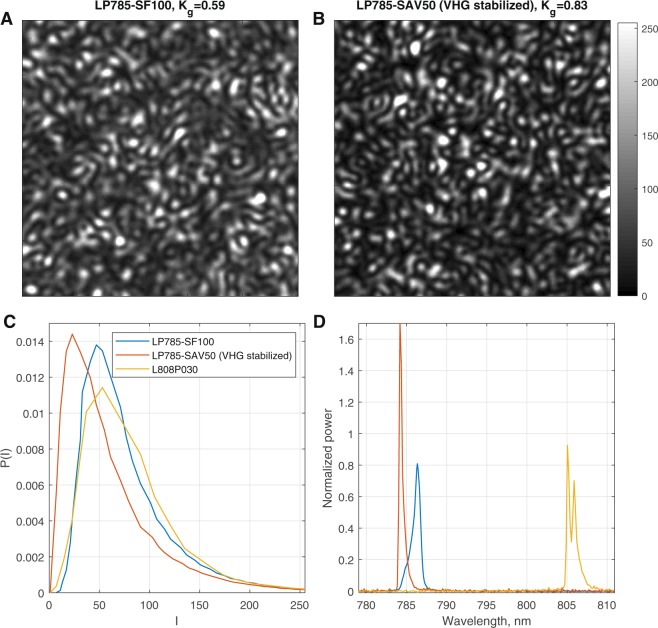
Static speckle patterns imaged from a piece of paper. (**A**,**B**) Patterns recorded with a conventional and a VHG stabilized laser diode respectively from the same spot on a piece of a thick white paper. (**C**) Pixel intensity distributions for three Representative lasers (LP785-SAV50, LP785-SF100, and L808P030). (**D**) Measured light spectrum of corresponding diodes. The intensity distribution recorded with the VHG laser is closer to the expected exponential decay and has a smaller offset than the conventional laser diodes. The improved performance of the VHG laser is also reflected in the global contrast *K*_*g*_ of 0.83 versus 0.59 for the conventional laser diode, and the more narrow spectrum.

There are no dynamics in the paper and the distribution of photon path lengths from the rough paper ensures a fully developed speckle pattern that will result in a speckle contrast of 1, provided source and detector properties are met. Typical speckle images and the corresponding intensity distributions obtained with the conventional non-stabilized laser diode LP785-SF100 and VHG stabilized LP785-SAV50 are shown in Fig. 1. It is evident that the speckle pattern recorded using the conventional laser diode appears to be blurred. Global speckle contrast was calculated to estimate the level of blurriness, using

1 $${K}_{g}=\frac{{\sigma }_{I}}{\langle I\rangle },$$

where 〈*I*〉 and *σ*_*I*_ are the mean value and the standard deviation of intensity calculated over all pixels of the image frame. Global contrast of the static speckle image was calculated for five laser diodes, resulting in *K*_*g*_ = 0.83 and 0.84 for the VHG stabilized diodes (LP785-SAV50 and LD785-SEV300) versus *K*_*g*_ = 0.59, 0.49 and 0.58 for the two LP785-SF100 and L808P030 non-stabilized diodes respectively. The intensity distributions and spectrum of the 3 representative lasers (LP785-SAV50, LP785-SF100, and L808P030) are compared in Fig. 1D revealing a difference in the spectral line width with the conventional laser diodes having a wider spectrum.

### Model

To explain the low values of observed contrast and verify to what degree the spectral properties of the light source are responsible for the reduced speckle contrast, we developed a model that combines speckle pattern generation and the imperfections introduced by camera related artifacts. We describe the measured electric field as a superposition of random waves following the procedure of^12^:

2 $$E(x,y,t)={\sum }_{j=1}^{N}{A}_{j}{e}^{i{k}_{xj}x+i{k}_{yj}y-i\omega {(\lambda ,\sigma )}_{j}t},$$

where the number of waves *N* = 1000, *A*_*j*_ is drawn randomly and uniformly from [0, 1], *ω* is drawn from a Gaussian distribution with mean corresponding to the simulated wavelength *λ* (785 nm) and standard deviation *σ* representing the spectral line width of the emitted light. The direction of the wave vector ${k}_{j}=\frac{w}{c}$, where *w* is the angular frequency of the light and *c* is the speed of light, is set to be random with a uniformly distributed polar angle *θ* ∈ [0..*π*], and azimuthal angle *ϕ* ∈ [0..2*π*]. *k*_*xj*_ and *k*_*yj*_ are calculated as *k*_*xj*_ = *k*_*j*_ cos(*θ*) sin(*ϕ*), *k*_*yj*_ = *k*_*j*_ cos(*θ*) cos(*ϕ*). The speckle pattern is the average intensity integrated over a time period corresponding to the exposure time *T*

3 $$I(x,y)={\langle |E(x,y,t){|}^{2}\rangle }_{t}.$$

**Figure 2:**
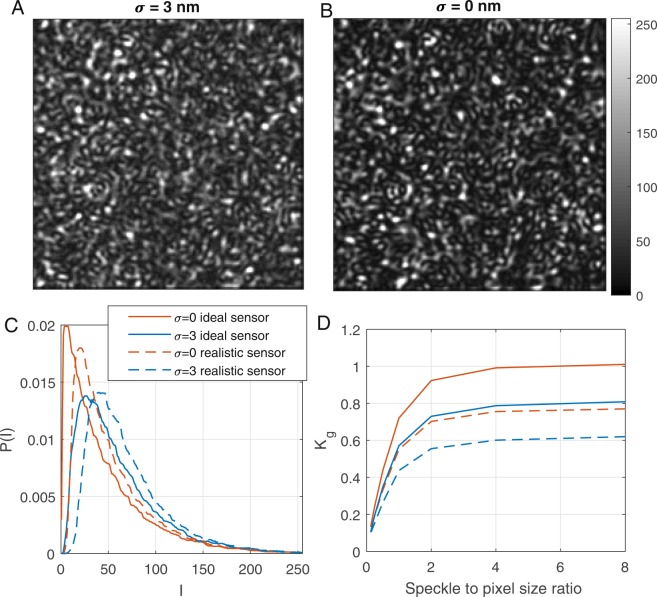
Model simulations. (**A**,**B**) Examples of speckle patterns for *σ* equal to 3 nm and 0 nm respectively. (**C**) Pixel intensity distribution. (**D**) Global contrast calculated for speckle patterns obtained with different speckle to pixel size ratio. For (**A**–**C**), the size ratio is equal to 4. It is evident that for larger spectral width that the speckle pattern becomes blurry and the contrast decreases, sensor imperfections decrease it even further.

We chose such way of modeling speckle dynamics instead of an established copula approach^13^ since it allowed us to incorporate a light source properties in a simple way. Exemplary spatial patterns and intensity distributions are shown in Fig. 2 with pixel size equal to *λ*/8, which corresponds to a speckle to pixel size ratio of 4. Similar to the experiment, the model with a more narrow light spectrum (i.e. smaller *σ* = 0) produced a sharper speckle pattern with a global contrast equal to 1.017 compared to 0.753 obtained with *σ* = 3 nm.

While the speckle pattern becomes more blurred with an increased spectral width, leading to a decrease of the global contrast (Fig. 2D, solid lines), it does not fully explain the lower contrast values of the static speckle pattern observed in our experiments (Fig. 1). To explore the issue further we have expanded the model to incorporate camera-related parameters that can affect speckle contrast including (i) rounding of the intensity value to an unsigned 8-bit integer, (ii) lateral pixel cross-talk^14^ and (iii) a fixed noise pattern. For simplicity, we implemented a symmetrical pixel cross-talk using a convolution of the speckle pattern with a 7 × 7 Gaussian kernel with a standard deviation of 0.5 pixels. The noise pattern was drawn from an exponential distribution, with a mean value of 11.8 estimated for the CMOS camera used in our experiments. We refer to simulations without these alterations as an “ideal sensor”, and simulations with all three of these parameters as a “realistic sensor”.

Introduction of these camera-related parameters led to a further decrease in speckle contrast, resulting in *K*_*g*_ = 0.788 when *σ* = 0 nm and *K*_*g*_ = 0.607 for *σ* = 3 nm. Interestingly, the relative contribution of the fixed noise pattern on *K*_*g*_ has the strongest effect when *σ* is low, and decreases as *σ* increases, while the effect of pixel cross-talk does the opposite. The effects of each camera parameter on *K*_*g*_ for different values of *σ* are elucidated in Table 1.

**Table 1:** Global contrast of the speckle patterns simulated with different spectral widths of the emitted light and different camera parameters introduced into the model.

*σ*, nm	*K*_*g*_ for different sensor models				
	Ideal	Rounding	Noise	Pixels cross-talk	Realistic
0	1.017	0.992	0.849	0.964	0.788
0.5	1.004	0.982	0.845	0.951	0.782
1	0.938	0.909	0.790	0.888	0.723
3	0.753	0.743	0.647	0.712	0.607
6	0.531	0.521	0.474	0.500	0.440
10	0.412	0.404	0.385	0.389	0.360

The speckle to pixel size ratio was set equal to 4.

### Flow imaging

**Figure 3:**
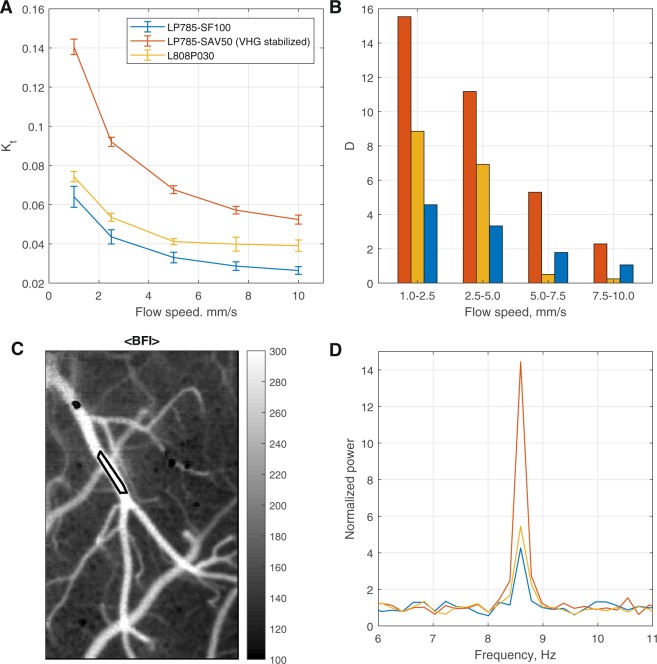
Flow imaging. (**A**,**B**) Phantom experiment. (**A**) Temporal contrast calculated for different flow speeds of Intralipid in the tube. (**B**) Corresponding detectability index (see Eq. 4). (**C**) Spatial contrast image recorded from the mouse brain. (**D**) The normalized power spectrum of cardiac pulsatility in the region marked by a red rectangle in (**C**). Using VHG stabilized laser results in a 2x increase  in the detectability index and ≈4 in the signal to noise ratio of the cardiac pulse recording.

To investigate the role of the light source spectral line width in the flow imaging and its effect on the signal-to-noise ratio (SNR), we have performed a dynamic phantom and *in-vivo* experiments. Results of the temporal contrast analysis applied to the phantom imaging are shown in Fig. 3A,B. From Fig. 3A one can make two important observations: first, the temporal contrast value *K*_*t*_ for the VHG laser is larger at any speed, and second, the corresponding standard deviation is either smaller or the same compared to the conventional lasers. This indicates that the VHG laser provides improved repeatability and a better signal to noise ratio.

In order to quantify the ability to distinguish changes in flow speed, we defined a “detectability index”:

4 $$D=|K({v}_{1})-K({v}_{2})|/(0.5\times ({\sigma }_{K}({v}_{1})+{\sigma }_{K}({v}_{2}))).$$

Here *σ*_*K*_ is the standard deviation of the measured *K*, *K* is the calculated speckle contrast value, and *v*_1_,*v*_2_ are two subsequent values of the Intralipid solution flow speed. This index thus quantifies the signal to noise ratio and permits comparison of the experimental results for the two different lasers. As shown in Fig. 3B, we see that the detectability index is 2 to 4 times greater for the VHG laser diode.

To demonstrate the effect of the improved signal to noise ratio *in vivo*, we measured the cardiac pulsatility signal in a cerebral pial artery of a mouse. The recorded data were processed to get local spatial contrast *K*_*s*_^2^ since it provides the high temporal resolution required to detect the cardiac pulsatility. The blood flow index was then calculated as $BFI(t)=\frac{1}{{K}_{s}{(t)}^{2}}$, where t is time, to estimate changes in flow. For the selected region of interest shown in Fig. 3C, the power spectrum of the *BFI* was calculated using a fast Fourier transform, which was then averaged and normalized by the median value of the noise plateau. Corresponding power spectrums for the different lasers are shown in Fig. 3D. It can be seen that the use of the VHG laser enhanced the signal to noise ratio by a factor of ≈4. While there is a difference between two conventional lasers it is negligible compared to the difference between stabilized and not stabilized diodes performance.

## Discussion

We have demonstrated that small changes in the spectral width of the light source can strongly affect the contrast of the speckle pattern. In practice, a volume holographic grating stabilized laser diodes (FWHM <0.5 nm) provides more than a 50% increase in contrast (*K*_*g*_ = 0.83 and 0.84 vs *K*_*g*_ = 0.59, 0.49 and 0.58) compared to the commonly used diodes. Modeling results show the same tendency, with *K*_*g*_ = 1.004 and 0.753 for *σ* equal to 0.5 and 3 respectively. Although the contrast decrease itself is not surprising, the actual values are. Earlier studies^9,10^ suggested that for light sources with coherence length longer than 100 microns one should not expect contrast below ≈0.8–0.9. In practice, however, a 3–5 times stronger reduction of the contrast is observed for the conventionally used laser diodes.

The contrast range is critical for LSCI applications, particularly in biomedicine. Sharper speckle patterns, provided by the use of the VHG lasers, improved detectability of the flow change by 2 to 10 times in a phantom experiment and improved the signal to noise ratio of cerebral cardiac pulsatility by a factor of ≈4. One advantage is that it will allow more detailed analysis of blood flow dynamics with less averaging, another advantage is to maintain a high SNR while using lower power density, which is crucial for application in the retina. Conventional semiconductor laser diodes have been used in most research and clinical applications of LSCI. Light sources with spectral width less than 1 nm should be used to achieve optimal SNR. Today, stabilized semiconductor laser diodes, i.e. diodes volume holographic grating stabilization, have become an affordable option.

Modeling camera related artifacts revealed the impact of noise and pixel cross-talk on the speckle pattern. While noise has a rather strong effect on contrast, it can be fully or partially compensated for during imaging. Pixel cross-talk, on the other hand, becomes more important considering the tendency towards smaller pixels and, consequently, stronger electric  cross-talk. This implies that oversampling the speckle by more than 2 pixels might be necessary to achieve better signal quality.

While laser speckle contrast imaging is the most obvious technology that suffers from blurred speckle pattern, narrowing of the spectral width will also lead to improved SNR in other techniques that are based on the analysis of speckle dynamics such as, for example, laser speckle rheometry^15^.

## Methods

A 1000 × 500 subset of pixels of a CMOS camera (Basler acA2040–180 kmNIR, 2048 × 2048 pixels, 4.4 *μ*m pixel size) was used to record the backscattered light, using a 5x objective with NA = 0.14 (Mitutoyo, Japan). The size of the speckle on the camera was adjusted by altering the pupil diameter of an iris in the detection path to achieve a speckle to pixel size ratio of approximately 4. A polarizer was placed in front of the objective. Coherent light was delivered to the object using a conventional semiconductor laser diodes (two different785 nm LP785-SF100 and 808 nm, L808P030, Thorlabs) or using a volume holographic grating (VHG) stabilized laser diodes (785 nm, LP785-SAV50, and LD785-SEV300, Tholrabs) which have a more narrow spectral line width resulting in a longer coherence length. Depending on the experiment either all five or three lasers were used. A CCD spectrometer (CCS175, Thorlabs) was used to measure the spectrum of the laser diodes with a 10 *μ*s integration time. The lasers were operated with the recommended settings. The optical power density on the object was controlled by expanding the size of the beam aiming for uniform illumination of the object with 0.5% of pixels to be saturated.

Static speckle patterns were recorded by illuminating a thick piece of rough paper using five different laser diodes at the 2 ms exposure time. Dynamic phantom was using a syringe pump moving a 10% solution of Intralipid through a polyethylene tube (0.28 mm inner diameter) at 1.0, 2.5, 5, 7.5 and 10 mm/sec. At each pump speed, the speckle pattern was recorded for 10 seconds, at 190 frames per second, with an exposure time of 5 ms. The tube was digitally masked in the image in order to avoid image analysis artifacts and for each pixel, the local temporal contrast *K*_*t*_ was calculated using 25 consecutive frames^16^. The 70 non-overlapping sequences of *K*_*t*_ values were used to estimate the mean contrast and standard deviation for each pumping speed.

Animal imaging was performed to test the observed effect *in-vivo*. All animal procedures were approved by the Boston University Institutional Animal Care and Use Committee and were conducted following the Guide for the Care and Use of Laboratory Animals. A chronic cranial window surgery was performed on a female B6C3 mouse (12-week-old, The Jackson Laboratory), as reported previously^17^. Briefly, a 3 × 3 mm craniotomy was drilled over the left somatosensory cortex, keeping the dura intact. A glass plug (made by fusing three 3-mm coverslips with one 5-mm coverslip) was inserted into the craniotomy, gently touching the brain. The plug was fixed with dental acrylic, along with an aluminum bar attached to the skull for head fixation during imaging. For this study, the animal was imaged approximately 16 weeks after surgery, under isoflurane anesthesia (3% induction, 1–1.5% maintenance, in oxygen), by fixing the head to a custom-made stage. Camera settings were the same as in the phantom experiments. Heart rate and oxygen saturation were noninvasively monitored (Mouse Stat Jr, Kent Scientific) to ensure that physiological conditions were maintained. The animal was allowed to recover after the experiment. Blood flow was recorded for 35 seconds with each representative laser using the same framerate and exposure time as in the phantom experiment. The spatial contrast was calculated using the 5 × 5 pixel’s neighborhood.

For both phantom and animal experiments, 3 lasers were tested: LP785-SAV50, LP785-SF100, and L808P030.

## Data Availability

The datasets generated during and/or analyzed during the current study are available from the corresponding author on reasonable request.
